# Suitable reference gene for quantitative real-time PCR analysis of gene expression in gonadal tissues of minnow *Puntius sophore* under high-temperature stress

**DOI:** 10.1186/s12864-017-3974-1

**Published:** 2017-08-15

**Authors:** Arabinda Mahanty, Gopal Krishna Purohit, Sasmita Mohanty, Nihar Ranjan Nayak, Bimal Prasanna Mohanty

**Affiliations:** 1 Fishery Resource and Environmental Management Division, ICAR- Central Inland Fisheries Research Institute, Barrackpore, Kolkata, 700120 India; 2 School of Biotechnology, KIIT University, Bhubaneswar, Odisha 751024 India; 3 Wayne State University Perinatal Initiative, Department of Obstetrics and Gynecology, Wayne State University School of Medicine, Detroit, MI 48201 USA

**Keywords:** Reference genes, RT-qPCR, Reproductive physiology, Thermal stress, *Puntius sophore*

## Abstract

**Background:**

High ambient temperature is known to affect fish gonadal development and physiology in a variety of ways depending on the severity and duration of exposure; however, the underlying molecular mechanisms are poorly understood. Gonadal gene expression influence the gonadal development, physiology and the quality of egg/sperm produced in teleosts and the mechanistic understanding of spatio-temporal changes in the gonadal gene expression could be instrumental in controlling the fate of egg/sperm and the quality of seed produced. Real time-quantititative polymerase chain reaction (RT-qCR), is a high throughput, sensitive and reproducible methodology used for understanding gene expression patterns by measuring the relative abundance of mRNA transcripts. However, its accuracy relies upon a suitable reference gene whose expression levels remain stable across various experimental conditions. In the present study, we evaluated the suitability of ten potential reference genes to be used as internal controls in RT-qPCR analysis in gonadal tissues (ovary and testis) of minnow *Puntius sophore* exposed to high temperature stress for different time periods (7 days, 60 days). Expression analysis of ten different constitutively expressed genes viz. 18S ribosomal RNA (*18S rRNA*), beta actin (*βactin*), β-2 microglobulin (*b2mg*), eukaryotic elongation factor-1 (*eef1*), glyceraldehyde-3phosphate dehydrogenase (*gapdh*), glucose-6-phosphate dehydrogenase (*g6pd*), ribosomal binding protein L13 (*rpl13*), tubulin (*tub*), tata box binding protein (*tbp*), ubiquitin (*ubi*) was carried out by using RT-qPCR and the stability in their expressions were evaluated by using four different algorithms; namely, delta Ct, BestKeeper, geNorm and NormFinder.

**Results:**

In ovary, *eef1* was found to be the most suitable reference gene in all the algorithms used. In testis, *b2mg* was found to be the most suitable reference gene in delta Ct, BestKeeper, NormFinder analysis while *tbp* and *eef1* were found to be the most suitable reference genes in geNorm analysis.

**Conclusions:**

In conclusion, *eef1* and *b2mg* were found to be the most suitable reference genes in ovary and testis, respectively, of *Puntius sophore* exposed to high temperature stress, and could be used as internal controls for gene expression analysis in gonadal tissues of *Puntius sophore*.

## Background

Gonadal gene expression influence sex determination, growth, and survivability of gametes in teleosts [1]. The ability to fully control sexual maturation, spawning and production of high quality seeds are some of the primary requirements for the successful development of aquaculture and in depth understanding of gene expression mechanism and the ability to manipulate it with high precision are important aspects of the reproductive physiology and biochemistry [2]. The ability to regulate sexual differentiation, maturation and reproduction provides fish breeders control over breeding processes thereby affecting large-scale industrial production [3]. The quality of fish gametes are determined by expression of an array of genes influenced by several factors (age, management, feeding, chemical and physical factors, water quality, etc.) that have an impact on the survivability of embryos, larvae and/or fry [4]. In recent times, fish gonadal gene expression studies are being used to assess the sperm/egg quality. Thus, gonadal gene expression studies have profound implications on the fisheries and aquaculture.

Gonadal gene expression in fish is affected by environmental factors such as water temperature, pH, dissolved oxygen content etc. [3]. Temperature is an important environmental factor that influences the sex ratio, quality of eggs and can have long lasting effects [5, 6] and increase in global temperature has been a cause of concern in the climate change regime [7, 8]. As fish exhibit enormous diversity in their morphology, habitat occupancy, and biology, it is necessary to extensively study the impacts of external temperature on the overall physiology and in the reproductive physiology in particular to assess species that will adapt to the changing environmental temperature and studying the gene expression pattern of fish species would be helpful in carrying out such assessments.

Gene expression study using quantitative real time polymerase chain reaction (RT-qCR), has become a high throughput, sensitive and reproducible methodology for measuring the relative abundance of mRNA transcripts and has been one of the most widely used method of gene expression analysis due to its accuracy, fastness, efficiency, sensitivity, reproducibility and broad dynamic range [9, 10]. However, its accuracy relies upon a suitable reference gene whose expression levels remain stable across various experimental conditions. In recent times, extensive effort are being made to find suitable reference genes that maintain constant expression levels under different experimental conditions [10–12]. However, no single gene has been found to have constant level of expression in all fish species, different tissues and experimental conditions. For instance, *18S rRNA*, *b2m* and *elfa* have been found to be suitable reference genes in gonadal tissues of zebrafish (*Danio rerio*) at different developmental stages whereas *rpl7* has been found to be the most suitable reference gene in gonadal tissues of *Oryzias latipes* [13, 14]. Therefore, it is necessary to identify species and organ specific reference genes that could be used for studies on transcriptomic responses of organisms under various conditions. In this context, the objective of the present study was to identify suitable reference gene(s) that can be used for RT-qPCR analysis in gonadal tissues (both ovary and testis) of minnow *Puntius sophore* exposed to high temperature stress for different time periods, both short term (7 days) and for long term (60 days). *Puntius sophore* is a close relative of *Danio rerio*, a widely used animal model and is a nutrient-dense small indigenous fish that is especially rich in micronutrients. It is available in most of the freshwater aquatic ecosystems in many tropical and subtropical countries like India, China, Bangladesh, Thailand, and Myanmar [15, 16] and owing to its high nutritive value, attempts are being made to bring it into aquaculture fold [17, 18]. The study identified suitable candidate reference genes in gonadal tissues of *Puntius sophore* which could be useful in evaluating different aspects of its reproductive physiology.

## Results

### Expression profiles of reference genes

Real-time reverse transcription quantitative polymerase chain reaction (RT-qPCR) is one of the most reliable methods of measuring transcript abundance, representing the spatio-temporal and clinical changes in gene expression. However, the results of RT-qPCR are very much dependent on the normalization of data against stable endogenous controls, often referred as housekeeping genes. Each of the sample was measured in triplicate for each run and nine independent replicates were performed for each set of experimental set up (Control, 7 days and 60 days heat exposed groups).

The expression levels of the candidate reference genes were calculated with respect to mean real-time PCR quantitative cycle (Cq) value. The Cq values of the candidate reference genes in ovary and testis tissues are presented in Table 1. In ovary tissue, *18S rRNA*, *βactin* were found to be the highly expressed genes. *gapdh* and *tbp* were found to be least expressed genes in ovary (Cq values ranging between 31 and 36 and 33–35 respectively. Similarly in testis, *βactin, rbpl13* and *18S rRNA* were found to be the highly expressed genes (Cq values ranging from 10 to 16, 15–23 and 15–24 respectively) whereas *g6pd*, *ubi* and *gapdh* were the least expressed genes.

**Table 1:** Cq values of different candidate reference genes in ovary and testis tissues of *Puntius sophore* (control and heat stressed for 7/60 days)

	*eef1*	*b2mg*	*18S rRNA*	*rbpl13*	*gapdh*	*βactin*	*tub*	*tbp*	*ubi*	*g6pd*
Ovary										
Control	20.49 ± 0.14	21.15 ± 0.05	14.36 ± 0.09	16.43 ± 0.3	36.65 ± 0.12	16.48 ± 0.22	17.39 ± 0.12	33.02 ± 0.03	14.76 ± 0.16	19.85 ± 0.27
7 days heat stressed	20.43 ± 0.17	21.59 ± 0.30	15.48 ± 0.30	21.40 ± 0.17	37.44 ± 0.23	15.74 ± 0.08	16.61 ± 0.18	33.39 ± 0.08	29.31 ± 0.08	19.75 ± 0.05
60 days heat stressed	20.44 ± 0.18	22.37 ± 0.10	11.39 ± 0.26	25.40 ± 0.17	31.51 ± 0.13	10.12 ± 0.03	16.36 ± 0.33	35.42 ± 0.21	18.59 ± 0.35	17.23 ± 0.08
Testis										
Control	23.47 ± 0.31	25.62 ± 0.25	20.57 ± 0.30	15.60 ± 0.15	36.45 ± 3.42	16.65 ± 0.22	23.72 ± 4.70	28.66 ± 0.18	38.63 ± 0.17	35.62 ± 0.26
7 days heat stressed	23.78 ± 0.10	25.41 ± 0.15	15.43 ± 0.15	17.78 ± 0.19	30.38 ± 0.10	10.26 ± 0.13	26.85 ± 0.19	27.36 ± 0.18	26.50 ± 0.12	38.54 ± 0.20
60 days heat stressed	20.50 ± 0.21	25.90 ± 0.63	24.57 ± 0.12	23.59 ± 0.13	32.78 ± 0.42	14.38 ± 0.09	26.38 ± 0.23	26.82 ± 0.12	38.00 ± 0.19	38.69 ± 0.32

### Gene expression stability of tissue specific reference genes due to thermal stress

In order to find out the suitable reference genes for normalization of RT-qPCR data, the stability of expression of ten candidate reference genes, across the control and heat stressed groups in gonadal tissues (ovary and testis) were measured and validated by using four computational methods, such as: comparative ΔCt, geNorm, NormFinder and BestKeeper [19–22]. These four programs, use different approaches in evaluating stability in gene expression and thus are used in combination to increase confidence over the results obtained.

### Comparative ΔCt method

**Fig. 1:**
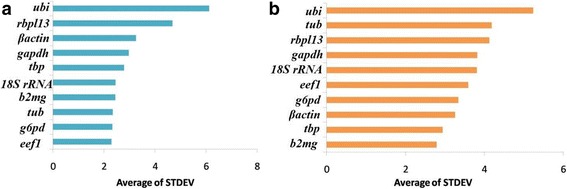
Stability in expression of 10 reference genes in **a** ovary and **b** testis of *Puntius sophore* as determined by delta Ct method. *eef1 and b2mg* were found to be the most suitable reference genes in ovary and testis, respectively

Comparative delta Ct method compares relative expression of a pair of genes; if the ΔCt value between the two genes remains constant when analyzed in different samples, it means both the genes are stably expressed among those samples. However, if the ΔCt fluctuates, then 1 or both genes are variably expressed. Then expression profiles of other genes are taken into consideration to find out the most stably expressed gene [19]. In the present study, *eef1* and *b2mg* were found to be most stably expressed genes in ovary and testis respectively with lowest average standard deviation values. The ranking of other candidate genes for suitability as a reference gene has been provided in Fig. 1.

### geNorm analysis

**Fig. 2:**
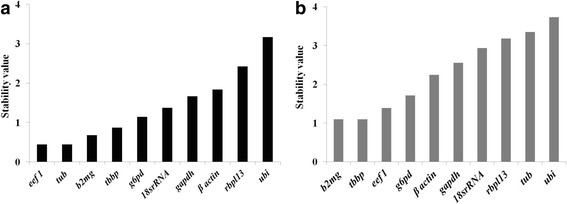
geNorm stability values of the 10 reference genes in **a** ovary and **b** testis of *Puntius sophore.* In ovary, *eef1 and tubulin* were the most stable genes whereas in testis *b2mg* and *tbp* were the most suitable reference genes. geNorm stability values (M) for each genes was calculated as the average pairwise variation for that gene with all other tested reference genes using the genorm software and genes were ranked in the increasing order of stability values

The geNorm software is employed as a mean for determining the expression stability value (M) for each candidate gene on the basis of the average pair wise variation between all genes analysed [10, 20]. The gene with the lowest M value is considered to have the most stable expression, while with the highest M value has the least stable expression. All the tested genes were found to be having high stability and stability values were within the geNorm cut off value (1.6) [23] except for *βactin*, *rbl1* and *ubi.* In testis *b2mg*, *tbp* and *eef1* were the genes which had stability values within the cutoff limit. geNorm analysis indicated that, the *eef1 and tubulin* were the most stable genes in ovary while *b2mg*, *tbp* were the most stable genes in testis (Fig. 2).

### NormFinder analysis

**Fig. 3:**
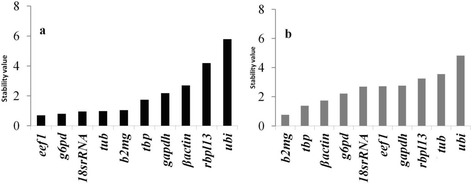
Normfinder stability values of the 10 reference genes in **a** ovary and **b** testis of *Puntius sophore.* In ovary, *eef1* was the most stable gene whereas in testis *b2mg* and *tbp* were the most suitable reference genes as determined by the Normfinder algorithm. Normfinder stability values were calculated by combining the inter and intra group variations determined using the excel-Normfinder add-in. The genes were then ranked in the increasing order of stability values

The NormFinder estimates the variance in the Cq value dataset, ranks the genes according to their stability. The more suitable gene expression is indicated by lower average expression stability values [21]. As per the NormFinder analysis, the order of stability in ovary was *eef1 > tubulin > b2mg > tbp > g6pd > 18S rRNA > gapdh > βactin > rbpl13 > ubi*. Similarly, in testis the order was *tbp > βactin > g6pd > 18S rRNA > eef1 > gapdh > rbl13 > tubulin > ubi* (Fig. 3).

### BestKeeper analysis

BestKeeper identifies the most suitable reference gene using the coefficient of variance and the standard deviation of the Cq values [22]. Candidate genes from all the samples were grouped in the following order, from most stable to least stable respectively; In ovary, *eef1 > tubulin > b2mg > tbp > g6pd > 18S rRNA > βactin > rbpl13 > ubi* and in testis, *b2mg > tbp > g6pd > eef1 > tubulin > gapdh > βactin > rbpl13 > 18S rRNA> ubi* (Table 2).

**Table 2:** Descriptive statistics and expression level of reference genes in (a) ovary and (b) testis as obtained by BestKeeper

a. Ovary										
	*18S rRNA*	*rbpl13*	*gapdh*	*ß-actin*	*tubulin*	*tbp*	*b2mg*	*ubi*	*eef1*	*g6pd*
n	81	81	81	81	81	81	81	81	81	81
geo Mean [CP]	13.62	20.75	35.1	13.79	16.78	33.92	21.7	20.03	20.45	18.91
AR Mean [CP]	13.73	21.08	35.2	14.11	16.79	33.94	21.7	20.89	20.45	18.95
min [Cq]	11.09	16.14	31.34	10.09	16.12	32.89	21.08	14.55	20.28	17.14
max [Cq]	15.88	25.52	37.67	16.67	17.49	35.61	22.51	29.42	20.68	20.18
Std. dev [+/− Cq]	1.58	3.09	2.46	2.66	0.42	0.98	0.51	5.62	0.15	1.14
CV [% Cq]	11.5	14.68	6.99	18.86	2.49	2.9	2.33	26.89	0.75	6.03
b. Testis										
	*18S rRNA*	*rbpl13*	*gapdh*	*ß-actin*	*tubulin*	*tbp*	*b2mg*	*ubi*	*eef1*	*g6pd*
n	81	81	81	81	81	81	81	81	81	81
geo Mean [Cq]	19.83	18.7	33.08	13.49	25.43	27.6	25.76	33.88	22.53	37.59
AR Mean [Cq]	20.19	18.99	33.21	13.76	25.65	27.61	25.76	34.38	22.58	37.61
min [Cq]	15.2	15.32	30.17	10.09	17.39	26.71	24.69	26.26	20.12	35.12
max [Cq]	24.94	24	53.47	16.86	27.65	28.98	27.32	38.94	24.01	39.43
Std. dev [+/− Cq]	3.18	3.06	2.18	2.34	1.96	0.7	0.4	5.25	1.39	1.33
CV [% Cq]	15.74	16.14	6.55	16.98	7.64	2.53	1.56	15.27	6.14	3.53

### Recommended comprehensive ranking

Combining the results of the analysis by four different algorithms, a comprehensive ranking was generated by the online tool available at http://leonxie.esy.es/RefFinder/. According to this ranking *eef1* was found to be the most suitable reference gene in ovary while *b2mg* was found to be the most suitable reference gene in testis (Table 3).

**Table 3:** Ranking of different candidate reference genes as determined by 4 different algorithms delta CT. BestKepper, NormFinder, geNorm

a. Ovary										
Ranking Order (Better--Good--Average)										
Method	1	2	3	4	5	6	7	8	9	10
Delta CT	*eef1*	*g6pd*	*tubulin*	*b2mg*	*18S rRNA*	*tbp*	*gapdh*	*βactin*	*rbpl13*	*ubi*
BestKeeper	*eef1*	*tubulin*	*b2mg*	*tbp*	*g6pd*	*18S rRNA*	*gapdh*	*βactin*	*rbpl13*	*ubi*
Normfinder	*eef1*	*g6pd*	*18S rRNA*	*tubulin*	*b2mg*	*tbp*	*gapdh*	*βactin*	*rbpl13*	*ubi*
geNorm	*eef1*	*tubulin*	*b2mg*	*tbp*	*g6pd*	*18S rRNA*	*gapdh*	*βactin*	*rbpl13*	*ubi*
Recommended comprehensive ranking	*eef1*	*tubulin*	*G6PD*	*b2mg*	*18S rRNA*	*tbp*	*gapdh*	*βactin*	*rbpl13*	*ubi*
b. Testis										
Ranking Order (Better--Good--Average)										
Method	1	2	3	4	5	6	7	8	9	10
Delta CT	*b2mg*	*tbp*	*ßactin*	*g6pd*	*eef1*	*18S rRNA*	*gapdh*	*rbpl13*	*tubulin*	*ubi*
BestKeeper	*b2mg*	*tbp*	*g6pd*	*eef1*	*tubulin*	*gapdh*	*ßactin*	*rbpl13*	*18S rRNA*	*ubi*
Normfinder	*b2mg*	*tbp*	*ßactin*	*g6pd*	*18S rRNA*	*eef1*	*gapdh*	*rbpl13*	*tubulin*	*ubi*
genorm	*tbp | eef1*		*b2mg*	*g6pd*	*ßactin*	*gapdh*	*18S rRNA*	*rbpl13*	*tubulin*	*ubi*
Recommended comprehensive ranking	*b2mg*	*tbp*	*eef1*	*g6pd*	*ßactin*	*gapdh*	*18S rRNA*	*tubulin*	*rbpl13*	*ubi*

## Discussion

Gonadal gene expression studies in fish are being employed to assess the quality of egg, spermatid, which in turn has profound implication in fish culture and management systems [5]. The control of gamete quality is of great importance; availability of good quality male and female gametes is necessary to close the lifecycle of a species and obtain subsequent generations and poor egg quality can lead to several types of problems [2]. It is also being used to understand developmental processes, and to assess the environmental effects on reproductive physiology of fish [24].

The RT-qPCR has emerged as a reliable, reproducible and highly sensitive technique for gene expression analysis. However, the reliability of the results largely depend on the selection of reference gene used for normalization of gene expression across different experimental conditions. In this regard, we have evaluated the stability of ten commonly used reference genes using Excel based algorithms deltCt, geNorm, NormFinder and BestKeeper. The results of analysis in all the algorithms used were very similar i. e. in ovary *eef1* was found to be the most stable gene in all the algorithms used. *eef1* performs varieties of functions during cell growth and proliferation, and plays key role in cytoskeleton organization, mitotic apparatus formation, and signal transduction [25–27]. It also mediates the recruitment of aminoacyl-tRNA to the A-site of the 80S ribosome during protein synthesis and thus is expressed ubiquitously in all cells [25, 26]. Similar to the present study, *elf1* has been found to be a suitable reference gene in gonadal tissues of Zebrafish exposed to endocrine disruptors [28] and *elfaα* has been found to be a suitable reference gene in gonads of rice field eel *Monopterus albus* in different stages of development [29]. Another translation related protein *eif* (eukaryotic initiation factor) has been found to be one of the most suitable reference genes in ovarian tissues of *Procambarus clarkii* [30].

Similarly in testis, *b2mg* was found to be the most stable gene in deltCt, NormFinder and BestKeeper algorithms. However, in geNorm analysis *tbp* and *eef1* were found to be the most stable genes. *b2mg* is a component of MHC class I molecules, which are present on all nucleated cells and it has been found to be a suitable reference gene in mouse hematopoetic stem cells [31], and in K562 cells and leucocytes of normal individuals as well as of malignoma patients [32] and different tissues of Japanese flounder (*Paralichthys olivaceus*) [33]. However, in some other studies involving human colorectal cancer cells [34] and human reticulocyte cells [19] it has been reported not to be a suitable reference gene.

The statistical algorithms used in the present study have distinguished features and thus there could be discrepancies between the results obtained. Therefore, these algorithms are used in combination to increase confidence limits on the results obtained from them. However, in the present study, we obtained same results from all the algorithms used except for the geNorm analysis in testis in which *tbp* was found to be the most stable gene in contrast to *b2mg* which was found to be the most stable gene in all the algorithms used.

## Conclusion

In conclusion, *eef1* and *b2mg* were found to be the most suitable reference genes in ovary and testis of *Puntius sophore* exposed to high temperature stress and can be used as internal controls for gene expression analysis in *Puntius sophore*. Identification of these reference gene would be helpful in carrying out gene expression analysis in gonadal tissues of *Puntius sophore*.

## Methods

### Ethical statement

The study, including sample collection, experimentation and sacrifice met the ethical guidelines, including adherence to the legal requirements of the study country. The study was approved by the Institute Animal Ethics Committee (IEAC) of ICAR-CIFRI vide approval no. CIFRI/IEAC-16-17/03.

### Sample collection after short and long term thermal exposure

*Puntius sophore* (weight: 4.22 ± 0.5 g, length: 6.46 ± 0.56 cm) were collected from aquaculture ponds and were acclimatized under laboratory conditions for 30 days. Fishes were kept in glass aquariums of 30 l capacity with temperature control systems. Fish were fed once daily @ 2% of body weight by providing laboratory prepared fish diet.

The fishes were randomly assigned to three experimental groups; control (acclimation temperature 27 ± 0.2 °C), fish exposed to heat stress (36 °C) for 7 days and fish exposed to heat stress for 60 days. The temperature in the heat stressed groups were initially increased at rate of 2 °C/h using the temperature control system attached with the aquarium and maintained at 36 ± 0.4 °C for the specified time period (7/60 days). 3/4th of the water was changed daily in each aquarium and were replenished with water of specified temperatures (27 ± 0.2 °C in control groups and 36 ± 0.4 °C for the heat stressed groups). After completion of the exposure period, fishes were dissected (after euthenization with Tricaine, MS-222, 200 mg/l) and ovary, testis tissues were collected in RNA later (R0901, Sigma) and were kept at −40 °C for further use.

### RNA extraction and cDNA synthesis

RNA was extracted from tissue samples (9 samples from each experimental group, weighing approximately 50–70 mg of tissues) using RiboZol (Himedia, India) according to the manufacturer’s protocol. Following isolation of RNA, the concentration of each RNA sample was measured by Bioanalyzer (Agilent 2100 Bioanalyzer, USA). RNA integrity was confirmed by denaturing agarose-formaldehyde gel (1% *w*/*v* agarose, 16% formaldehyde) electrophoresis showing two sharp and intense bands for 18S & 28S ribosomal RNA. RNA samples were treated with the DNase1 (NEB, UK) as per the manufacturer’s recommended protocol to remove DNA contamination. 1 μg of DNase treated total RNA was reverse transcribed using M-MLV reverse transcriptase (NEB, UK) according to manufacturer’s protocols. The cDNA so obtained were diluted 10 fold with nuclease free water for further amplification in RT-qPCR.

### Primer synthesis

Ten candidate reference genes were evaluated in the present study such as *βactin*, *gapdh, rbpl13*, *tub*, *18S rRNA*, *tbp, b2mg, eef1, g6pd, ubi*. Primers for *βactin*, *gapdh, rbpl13, tub, 18S rRNA*, and *tbp* were adapted from Purohit et al. (2016) [10]. Primers for *b2mg, eef1, g6pd* were adapted from McCurley and Callard [13] and those for *ubi* were adapted from Olsvik et al. 2008 [35]. Primers with specific nucleotide sequences were obtained from Integrated DNA Technologies, USA. The specificity of the primer sets were confirmed by the presence of a single band of appropriate size obtained after PCR amplification.

### RT-qPCR

The real time PCR amplifications were carried out using SYBR Green detection chemistry. cDNA were run in triplicates on a 96 well reaction plates with the CFX Connect real time PCR (Bio-Rad, UK). 20 μl of reaction mixture containing 10 μl of VeriQuest SYBR Green Mix (BioRad, UK), 1.0 μl of each 10 μM of primers and 5 μl of diluted cDNA as template and 3 μl RNase/DNase free sterile water (Thermo Scientific, USA).

The following amplification program were used in all RT-qPCR reactions: 50^0^ C for 2 min, 95 °C for 10 min, 40 cycles of 15 s 95 °C annealing, extension for 45 s at optimized temperatures for specific candidate genes (Table 4). The specificity of each amplification reaction was verified by a melting curve analysis after 40 cycles. No template controls (NTC) were included for each primer pair to avoid possible contamination of assay reagents and one negative control (without reverse transcription) per sample to identify residual gDNA after DNase digestion.

**Table 4:** Candidate genes, primer pairs and different q-PCR parameters

Gene symbol	Primer Forward	Primer Reverse	Amplicon Size	Tm	Fish species	Accession no.	PCR Efficiency (%)	Reference
*18S rRNA*	GGTAGACACACGCTGATCCA	CATGGCCGTTCTTAGTTGGT	96	60	*Channa striatus*	KC800799	99.1	Purohit et al. 2015 [10]
*βactin*	GCCTTCCTTCCTTGGTATGG	GTGTTGGCGTACAGGTCCTT	89	59	*Channa striatus*	KC967219	98.50	Purohit et al. 2015 [10]
*b2mg*	GCCTTCACCCCAGAGAAAGG	GCGGTTGGGATTTACATGTTG	103	55	*Danio rerio*	BC062841	99.97	McCurley and Callard 2008 [13]
*tub*	CCTGCTGGGAACTGTATTG	TCAATGAGTTCCTTGCCAAT	89	54	*Channa striatus*	KC710731	97.64	Purohit et al. 2015 [10]
*eef1*	CTTCTCAGGCTGACTGTGC	CCGCTAGCATTACCCTCC	109	56	*Danio rerio*	AY422992	99.86	McCurley and Callard 2008 [13]
*gapdh*	ATCAAGGAAGCGGTGAAGAAGG	CGAAGATGGAGGAGTGGGTGTC	99	60	*Channa striatus*	KF044311	95.21	Purohit et al. 2015 [10]
*g6pd*	GTCCCGAAAGGCTCCACTC	CCTCCGCTTTCCTCTC	112	62	*Danio rerio*	BMI82602	98.65	McCurley and Callard 2008 [13]
*rbpl1*	ATGAACACCAACCCTTCCCG	GGTGGAGGGATGCCATCAAA	95	59	*Channa striatus*	KC915026	96.88	Purohit et al. 2015 [10]
*tbp*	CTTACCCACCAGCAGTTTAGCAG	CCTTGGCACCTGTGAGTACGATTTG	126	57	*Channa striatus*	KF736933	95.37	Purohit et al. 2015 [10]
*ubi*	GGCCGCAAAGATGCAGAT	CTGGGCTCGACCTCAAGAGT	69	58	*Gadus morhua*	EX735613	99.78	Olsvik et al. 2008 [35]

PCR efficiency of the reference genes were determined by a standard curve analysis of cDNA samples according to the MIQE guideline [36]. A series of 10 fold dilution of three replicates of cDNA, were made to determine the gene specific PCR amplification efficiency for each primer pair used in RT-qPCR experiments. Standard curve were derived from the E values by the formula E = 10^–1/slope^. The mean efficiency values were obtained for each tissue samples and were used to adjust the quantitative cycle (Ct) values for further analysis.

### Data analysis

Stability of mRNA expression of each candidate genes was statistically analyzed with four widely used software programs such as comparative Ct, geNorm, NormFinder and BestKeeper [19–22]. geNorm stability values (M) for each genes was calculated as the average pairwise variation for that gene with all other tested reference genes using the genorm software and genes were ranked in the increasing order of stability values. The Normfinder stability values were calculated by combining the inter and intra group variations determined using the excel-Normfinder add-in. The genes were then ranked in the increasing order of stability values. Descrptive statistics was carried out by SPSS 16.0 and Bestkeeper excel add-in. The comprehensive ranking of different genes were evaluated using the online resource available at http://leonxie.esy.es/RefFinder/ which assigns an appropriate weight to individual genes and calculates the geometric mean of their weights obtained for the overall final ranking [37].

## Abbreviations

RT-qPCR: Real time-quantititative polymerase chain reaction
